# Grazing Intensity Modifies Soil Microbial Diversity and Their Co-Occurrence Networks in an Alpine Steppe, Central Tibet

**DOI:** 10.3390/microorganisms13010138

**Published:** 2025-01-10

**Authors:** Mingxue Xiang, Zepeng Liang, Yanjie Zhang, Junxi Wu, Tao Ma, Lha Duo, Xianzhou Zhang, Gang Fu

**Affiliations:** 1 State Key Laboratory of Plateau Ecology and Agriculture, Qinghai University, Xining 810016, China; 2023990034@qhu.edu.cn (M.X.); matao2438@163.com (T.M.); 2 Lhasa Plateau Ecosystem Research Station, Key Laboratory of Ecosystem Network Observation and Modeling, Institute of Geographic Sciences and Natural Resources Research, Chinese Academy of Sciences, Beijing 100101, China; fugang@igsnrr.ac.cn; 3 School of Science, Tibet University, Lhasa 850011, China; 16675821426@163.com (Z.L.); lhaduo@hotmail.com (L.D.); 4 College of Agricultural and Biological Sciences, Dali University, Dali 671003, China; zhangyj@dali.edu.cn; 5 College of Resources and Environment, University of Chinese Academy of Sciences, Beijing 101408, China

**Keywords:** alpine grassland, co-occurrence network, diversity of bacteria and fungi, grazing intensity, Tibet

## Abstract

Grazing intensity is one of the crucial anthropogenic activities on alpine grasslands. However, how grazing intensity affects soil microorganism diversities and their co-occurrence networks in alpine steppe remains uncertain. We carried out a controlled grazing experiment (null grazing, CK; moderate grazing, MG; and heavy grazing, HG) on a typical alpine steppe in the Lhasa River Basin, Central Tibet, China. We used high-throughput sequencing to find the sequences of bacterial 16S rRNA and fungal ITS gene amplicons. Then, we analyzed their alpha and beta diversities and set up co-occurrence networks that show how often they occur together. MG significantly increased the bacterial Shannon index and changed the bacterial community structure. In contrast, HG decreased the fungal ACE and Chao1 indices and also changed the fungal community structure (*p* < 0.05). Linear mixed-effect model revealed that available phosphorus in soil significantly impacted on soil bacterial Shannon, ACE, and Chao1 indices across grazing intensities, while total carbon in subsoil significantly affected these indicators of soil fungi. Moreover, MG increased the complexity of the co-occurrence network in the bacterial community, while HG simplified it. However, both MG and HG made the co-occurrence networks in the fungal community less complicated. This shows that the intensity of grazing has different impacts on how microbes interact with each other. Therefore, sustainable grazing intensity necessitates a deeper understanding of biodiversity conservation in alpine grasslands.

## 1. Introduction

The Qinghai–Tibet Plateau (QTP) covers approximately 2.5 million km^2^, most of it occupied by alpine grasslands [1]. Researchers acknowledge the heightened ecological sensitivity of alpine grasslands, characterized by their elevated altitude and stringent environmental conditions, compared to other grassland ecosystems [2,3,4]. Grazing is the predominant land use in alpine grasslands [5]. Soil microorganisms are integral components of grassland ecosystems, and they play a pivotal role in sustaining ecosystem functions. They are instrumental in mediating key ecological processes, including the decomposition of organic matter, nutrient cycling, and the complex interactions among plants, soil, and other microorganisms [6]. However, our understanding of how soil microorganisms in alpine grasslands respond to grazing intensity is inadequate.

Microorganisms occupy a majority of spatial niches in the soil due to their high alpha (α) and beta (β) diversity and the tight associations within microbial groups—co-occurrence networks [7]. However, several factors influence the microbial diversity, including soil microtopography and microclimate [8], plant and soil characteristics [6,9], grazing activity [10,11], and grassland degradation and restoration processes [12]. Among these factors, grazing is considered the primary driver influencing soil microbial diversity [10,11]. The selective intake, trampling, and dung return induced by grazer could directly and/or indirectly influence the species composition and structure of a microbial community [13,14,15,16,17]. However, changes in microbial α- and β-diversity due to grazing are largely dependent on the grazing intensity. For instance, a recent meta-analysis suggests that light-to-moderate grazing can enhance the diversity of soil bacterial and fungal species in global grasslands [18]. Prolonged grazing alters the composition of the microbial community and the relative abundance of specific microbial groups within southern grasslands [19]. Light grazing increased soil microbial α-diversity, whereas moderate grazing increased soil microbial β-diversity in the meadow steppe [20]. Despite alpine grasslands’ higher sensitivity compared to other grasslands, our understanding of how different grazing intensities in alpine grasslands affect microbial diversity is insufficient.

The microbial co-occurrence network denotes the positive or negative associations between microbial groups, indicative of cooperative or competitive interactions among microorganisms [21,22]. Two mechanisms primarily mediate the effects of grazing on microbial co-occurrence networks: direct influences, such as selection processes, and indirect influences. Selection processes shape microbial community composition through both environmental filtering and species interactions [22,23]. Grazing, as one of the key environmental stresses in grasslands, directly affects the microbial aggregation and assembly that shape microbial co-occurrence networks [24,25]. Alternatively, changes in aboveground plants and the availability of nutrients caused by grazing indirectly cause changes in soil microbes and the networks that connect them [24]. Varying grazing intensities also indirectly influence the plant and soil properties within the grassland ecosystems, potentially leading to biased competition among microbial groups [26]. Soil or plant factors may constrain the dispersal of soil microorganisms, potentially affecting their growth rates [27] and the connectivity and complexity of microbial network modules [28]. However, the effect of grazing on microbial co-occurrence networks depends a lot on the environmental conditions [29]. This is true no matter how much grazing changes the characteristics of plants and soil. So, changes in the structures, interactions, and co-occurrence networks of soil microbes may be different depending on the level of grazing. Thus, it is imperative to further explore how different grazing intensities modify soil microbial co-occurrence networks within alpine grasslands.

According to the previous study, heavy grazing significantly decreased vegetation aboveground biomass and plant species diversity [30]. Thus, we hypothesize that heavy grazing decreases the soil microbial diversity and co-occurrence networks compared to null grazing and moderate grazing. We performed a fence-based grazing experiment in an alpine steppe of the Lhasa River Valley in Tibet, China, to explore (1) how different levels of grazing affect the α- and β-diversity and (2) how the co-occurrence networks of soil microbes (including bacteria and fungi) respond to grazing intensity. This observational site has been subject to three degrees of grazing intensity for 6 years: null grazing, moderate grazing, and heavy grazing, with five replicates per level [30]. Because of this long-term observational site, the many field surveys, soil sample collections, and high-throughput sequencing, we can learn more about how human activity affects the protection of soil biodiversity in alpine grasslands.

## 2. Materials and Methods

### 2.1. Site Description and Grazing Experimental Design

Established in May 2011, the experimental site is located in Linzhou County (29°52′ N, 91°07′ E), at an altitude of 3900 m, in the Tibet Autonomous Region of China. This site, located within the gravel basin of the Lhasa River, embodies a typical semi-arid alpine grassland. Its climate, characterized by a semi-arid temperate plateau monsoon climate, ranges from 10.6 to 12.6 °C during the growing season. Over 70% of annual precipitation occurs during the growing season, ranging from 343 to 466 mm. The WRB classification 2022 classifies the soil type at the site as Umbric Leptosols, with an average depth from 20 to 30 cm. The dominant plant species in this grassland include *Tripogon bromoides*, *Pennisetum centrasiaticum*, *Kobresia pygmaea*, and *Carex atrofusca* [30].

Initiated in 2016, the grazing experiment followed a typical fenced grazing design from 2015. A total of 15 fenced plots (measuring 10 m × 10 m) were established for three grazing intensities: null grazing (0 sheep ha^−1^ a^−1^, CK), moderate grazing (1.65 sheep ha^−1^ a^−1^, MG), and heavy grazing (2.47 sheep ha^−1^ a^−1^, HG), with 5 replicates for each grazing intensity. The livestock used in the study was Pengbo sheep, a native female breed of Tibetan sheep. The fenced grazing experiment was conducted monthly, from July to September; and annually, from 2016 to 2022 [30].

### 2.2. Soil Sampling

Soil samples were collected in July 2022 by a soil drill with an internal diameter of 3 cm. A 5-point sampling method was applied within 15 fenced plots [31,32]. We specifically selected five points in each fenced plot, drilled soil samples from a depth of 0–20 cm, and mixed them into a single composite sample to serve as the soil sample for the fenced plot [31,32]. We then submerged the soil samples in liquid nitrogen to analyze soil nutrient content and microbial community composition and diversity. It is essential to emphasize that the removal of surface vegetation and litter is necessary prior to conducting soil-sampling procedures. Furthermore, to prevent potential sampling errors, it is important to disinfect the soil drill with alcohol before and after each sampling event.

### 2.3. DNA Extraction, PCR Amplification and High-Throughput Sequencing

To extract DNA from soil microorganisms and sequence their amplified fragments, we initially used the MagPure Stool KF Kit B (MAGEN, Shenzhen, Guangdong, China) to obtain 30 ng DNA from freshly frozen soil stored at −80 °C. We then used the extracted DNA to construct bacterial 16S rDNA gene and fungal ITS gene amplicons using the fusion primer method. To ensure the quality and reproducibility of our sequencing data, we implemented the following quality control measures:

1. DNA concentration and purity Assessment: We precisely measured the sample concentration using Qubit and constructed a DNA library for only qualified samples, ensuring that the DNA concentration was adequate for subsequent steps.
2. Fragmentation and adapter ligation quality control: After DNA fragmentation, we verified that the fragment size distribution was within the desired range. We checked the efficiency of the adapter ligation process and confirmed that adapters were successfully incorporated.
3. PCR amplification and adapter dimer detection: Following PCR amplification, we verified that the amplification was successful without over-amplification or under-amplification, which can introduce biases and affect library complexity. We also monitored and detected the presence of adapter dimers, minimizing their content to ensure a high-quality library.
4. Read quality trimming: We set a window length of 30 bp and trimmed read ends if the average quality value within the window was below 20. We removed reads that were shorter than 75% of their original length after trimming, ensuring that only high-quality reads were included in the analysis.

To address potential issues of sequencing errors or chimera formation, we employed the following strategies:

1. Primer trimming with cutadapt v2.6: We used the software cutadapt v2.6 to remove primers and adapter contamination from the reads, ensuring that we obtained clean sequences of the target regions.
2. Quality trimming based on windowed approach: We employed a windowed approach where a 30 bp window was set. If the average quality value within this window was below 20, we trimmed the read from the start of the window. Additionally, any reads that were shorter than 75% of their original length after trimming were removed.
3. Removal of reads containing Ns: We also removed reads that contained ambiguous nucleotides (Ns) to prevent any uncertainty in the sequence data.
4. Removal of low-complexity reads: To address the issue of chimera formation, we removed reads with low complexity, specifically those with continuous sequences of 10 or more ATCG, which are prone to forming chimeras.

For the sequencing process, we employed the Illumina HiSeq platform (San Diego, CA, USA), which is known for its high-throughput sequencing capabilities and high data quality. Our target sequencing depth was set to ensure comprehensive coverage of the bacterial 16S rDNA gene and fungal ITS gene amplicons. On average, we achieved a sequencing depth of approximately 150 million paired-end reads per sample, which allowed for a thorough analysis of the soil microbial communities.

During the construction of the PCR system, the PCR mixture was prepared with 30 ng of DNA samples and fusion primers. The V3-V4 region of bacteria was amplified with the forward primer of 341F (5′-ACTCCTACGGGAGGCAGCAG-3′) and the reverse primer of 806R (5′-GGACTACHVGGGTWTCTAAT-3′) [32]. Similarly, the ITS1 region of fungi was amplified with the forward primer its1 (5′-CTTGGTCATTTAGAGGAAGTAA-3′) and the reverse primer its2 (5′-GCTGCGTTCTTCATCGATGC-3′) [31]. PCR amplification was performed following the specified reaction parameters. The amplified product was purified using Agencourt AMPure XP beads (Brea, CA, USA), dissolved in elution buffer, and then labeled, and a DNA library was prepared. Subsequently, the library was analyzed with the Agilent 2100 Bioanalyzer (Santa Clara, CA, USA) to determine the fragment range and concentration. Qualified libraries underwent high-throughput second-generation sequencing, and the low-quality reads were removed. The remaining high-quality, clean data were used for further analysis.

OTU Clustering Similarity Threshold: In the analysis of the sequencing data, we grouped the reads into Operational Taxonomic Units (OTUs) using a 97% sequence similarity threshold, which is a standard in the field and corresponds to the average level of 16S rRNA gene sequence divergence between different bacterial species. This threshold allowed us to define ecologically and evolutionarily meaningful taxonomic units.

Finally, the reads were merged into tags based on overlap, and species annotation was conducted on the OTUs by aligning them to the NCBI database. The Shenzhen BGI Gene Technology Co., Ltd. (Shenzhen, China), carried out all the above steps.

### 2.4. Data Analysis

For the obtained OTU list and its annotation information, the alpha diversity analysis of microorganisms was carried out according to the following procedures:

(1) Shannon index (*H’*):

$$H^{\prime }=-\sum P_{i}ln(P_{i}),$$

where *P_i_* is the ratio of the *i*th OTU number in the sample to the total OTU number.

(2) ACE index (*S_ACE_*):

$$S_{ACE}=S_{abund}+\frac{S_{rare}}{C_{ace}}+\frac{a_{1}}{C_{ace}}*\gamma^{2},$$

$$C_{ace}=1-\frac{a_{1}}{N_{\left\{rare\right\}}},$$

$$\gamma^{2}=max\left(\frac{\frac{S_{rare}}{C_{ace\left(sum\left[i=1\ldots 10\right]*a_{i}\right)}}}{\frac{N_{rare}}{N_{rare}-1}}-1,\;0\right),$$

where *a_i_* is the abundance of species *i*; *S_rare_* is the number of rare species; *S_abund_* is the number of abundant species, in which the abundance threshold of rare species is set to 10; and *N_rare_* is the number of individuals of rare species.

(3) Chao1 index (*S_Chao1_*):

$$S_{chao1}=S_{0}+\frac{a_{1}(a_{1}-1)}{2(a_{2}+1)}$$

where the OTU abundance of *a*_1_ species 1, *a*_2_ is the OTU abundance of species 2, and *S*_0_ represents the actual measured OTU numbers.

The beta diversity of microorganisms was analyzed to evaluate the community composition and similarity between the different grazing intensities. Principal coordinates analysis (PCoA) based on Bray–Curtis distance was used to reflect sample similarity with the “*vegan (2.6-6.1)*” package in R. Additionally, multivariate analysis, including permutational multivariate analysis (PARMANOVA) and adonis pairing tests, was performed to examine significant differences in microbial community similarity between the grazing intensities with the “*vegan (2.6-6.1)*” and “*pairwiseAdonis (0.4)*” packages in R. The Mantel test and linear mixed-effect model (LMM) were used to investigate the effects of plant diversity, biomass, soil nutrients, and soil pH on soil microorganisms. Network co-occurrence analysis was used to investigate microbial interactions (cooperation and competition) between bacteria and fungi at different grazing intensities with the “*psych (2.4.3)*” and “*igraph (2.0.3)*” packages in R.

All of the above analyses were performed using R (version 4.1.3 for Windows). The visualization microbial diversity and PCoA were performed in the R package “*ggplot2* (3.5.1)”. The visualization of microbial co-occurrence networks was conducted in Gephi (version 0.10.1 for Windows).

## 3. Results

### 3.1. Soil Microbial Diversity

**Figure 1:**
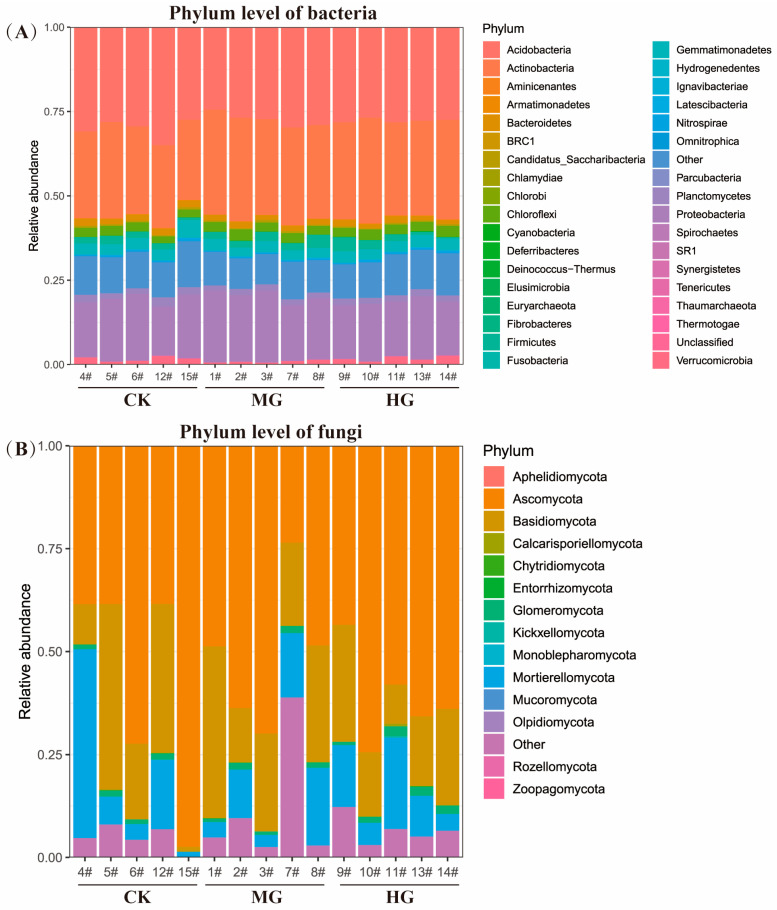
The compositions of soil bacteria (**A**) and fungi (**B**) at the phyla level among different grazing intensities. Null grazing, CK; moderate grazing, MG; and heavy grazing, HG.

*Acidobacteria*, *Actinobacteria*, and *Proteobacteria* were the top three dominant phyla of soil bacteria under three grazing intensities (Figure 1A). In addition, *Ascomycota*, *Basidiomycota*, and *Mortierellomycota* were the top three dominant phyla of soil fungi under three grazing intensities (Figure 1B). In contrast to soil bacteria, the dominant phylum of soil fungi exhibited considerable variation across grazing intensities. Moreover, the relative abundance of other bacterial and fungal phyla showed only minor fluctuations.

**Figure 2:**
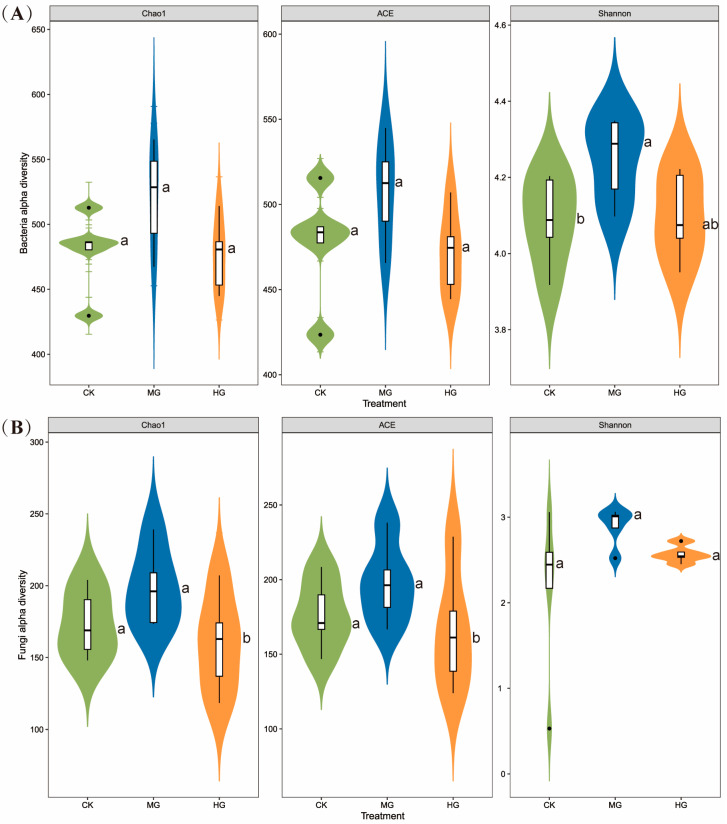
Soil bacterial (**A**) and fungal (**B**) community diversity indices (Shannon index, ACE index, and Chao1 index) among the different grazing intensities. Lowercase letters (a and b) indicate significant differences (*p* < 0.05) between three grazing intensities. Null grazing, CK; moderate grazing, MG; and heavy grazing, HG.

The Shannon index of soil bacteria significantly increased under MG conditions (Figure 2A, *p* < 0.05). However, the Chao1 and ACE indices of soil fungi decreased significantly under HG conditions (Figure 2B, *p* < 0.05). We observed no significant differences in the Shannon index of soil fungi or the Chao1 and ACE indices of soil bacteria between the different grazing intensities.

**Figure 3:**
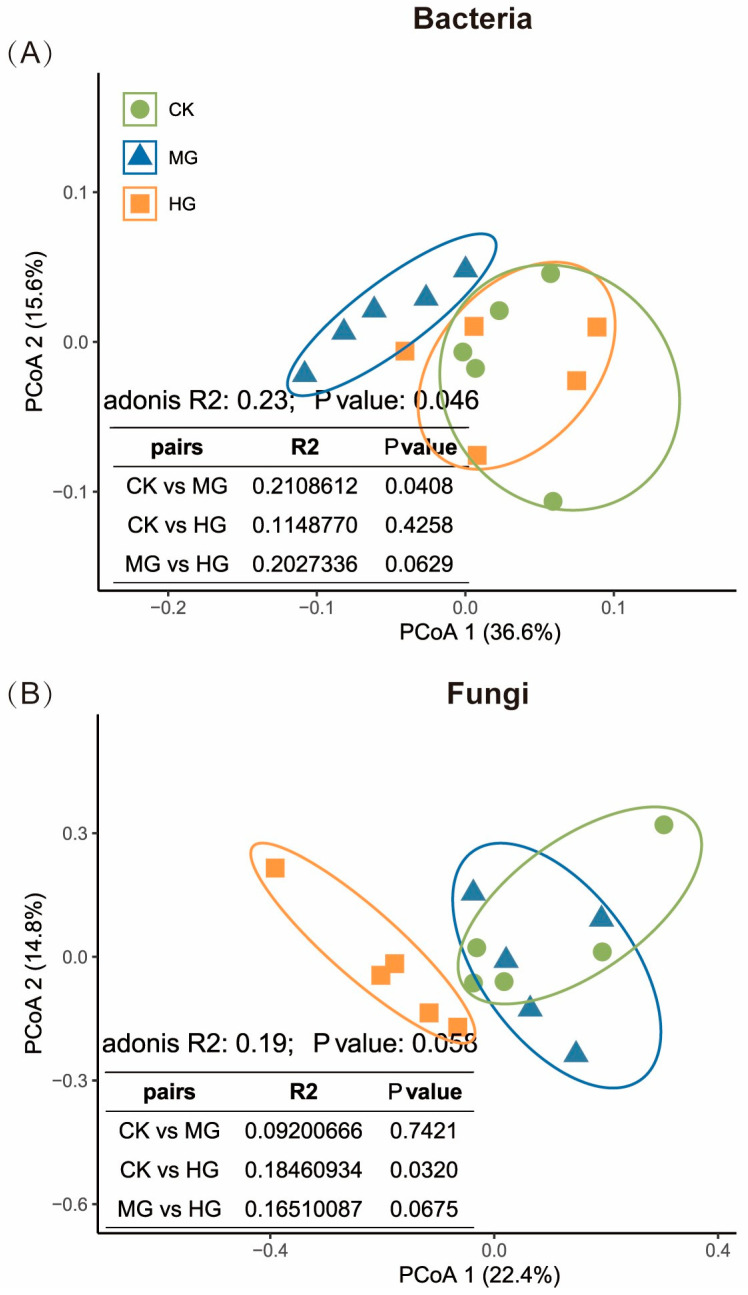
Principal coordinates analysis of the taxonomy-based dissimilarities and adonis pairing tests in soil bacterial (**A**) and fungal (**B**) communities at different grazing intensities. Null grazing, CK; moderate grazing, MG; and heavy grazing, HG.

Compared to the CK, MG significantly altered the soil bacterial community structure (Figure 3A, *p* = 0.041), while HG and CK showed no difference (Figure 3A, *p* = 0.426). In contrast, HG significantly altered the soil fungal community structure compared to the CK (Figure 3B, *p* = 0.032). MG and HG showed a marginal difference (Figure 3B, *p* = 0.068). MG and CK did not significantly differ in the soil fungal community (Figure 3B, *p* = 0.742).

### 3.2. Relationship Between Soil Microorganisms, Plants and Soil Nutrients

**Figure 4:**
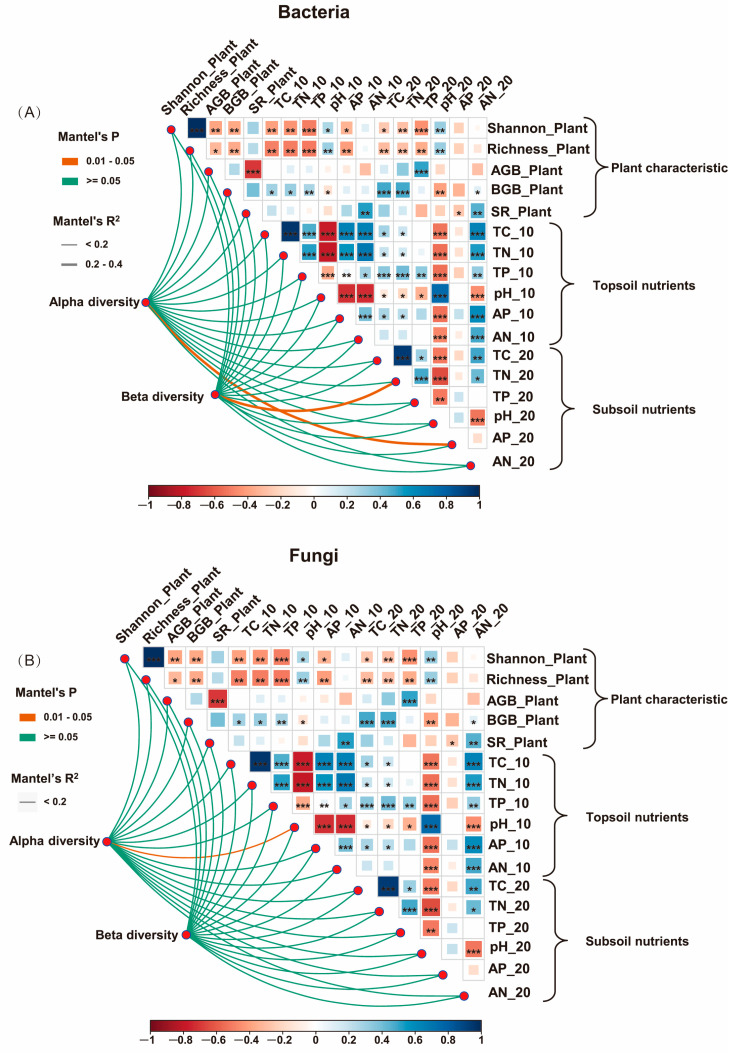
The Mantel test and correlation analysis of alpha and beta diversity of soil bacteria (**A**) and fungi (**B**) with plant diversity (Shannon diversity and richness) and soil nutrients (topsoil and subsoil). Shannon_Plant, Richness_Plant, AGB_Plant, and BGB_Plant indicate plant community Shannon diversity, richness, above-ground biomass, and below-ground biomass, respectively. Soil nutrients with ’_10’ and ‘_20’ indicate topsoil (0–10 cm soil depth) and subsoil (10–20 cm soil depth) nutrients, respectively. TC, soil total carbon; TN, soil total nitrogen; TP, soil total phosphorus; pH, soil pH; AP, soil available phosphorus; and AN, soil available nitrogen. The colored lines indicate the significance of the Mantel test results: orange, 0.01 < *p* < 0.05; green, *p* ≥ 0.05. Line thickness indicates correlation coefficient size. Asterisks indicate the significance effect: * *p* < 0.05, ** *p* < 0.01, *** *p* < 0.001.

The Mantel test and correlation analysis demonstrated a significant correlation between the alpha diversity of soil bacteria and the available phosphorus content in the subsoil (Figure 4, *p* < 0.05). Additionally, the beta diversity of soil bacteria was significantly correlated with soil total nitrogen (Figure 4A, *p* < 0.05). However, the alpha diversity of soil fungi was significantly correlated with topsoil pH (Figure 4B, *p* < 0.05).

**Figure 5:**
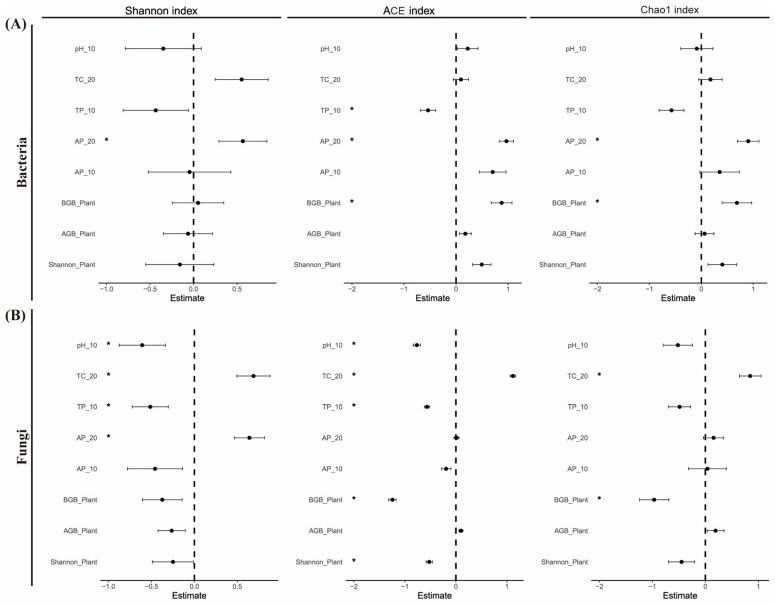
Linear mixed-effect model for controlling factors (Shannon index, above- and belowground biomass, and soil nutrients) of soil bacterial (**A**) and fungal (**B**) diversity indices. Shannon_Plant, AGB_Plant, and BGB_Plant indicate plant community Shannon diversity, above-ground biomass, and below-ground biomass, respectively. Soil nutrients with ‘_10’ and ‘_20’ indicate topsoil (0–10 cm soil depth) and subsoil (10–20 cm soil depth) nutrients, respectively. TC, soil total carbon; TP, soil total phosphorus; pH, soil pH; and AP, soil available phosphorus. Asterisks indicate the significance effect: * *p* < 0.05.

It was found that subsoil available phosphorus had a big effect on the Shannon, ACE, and Chao1 indices of soil bacteria, while belowground biomass (BGB) of plants had a big effect on the ACE and Chao1 indices of soil bacteria (Figure 5A, *p* < 0.05). Topsoil total phosphorus significantly influenced the ACE index of soil bacteria alone (Figure 5A, *p* < 0.05). Total carbon in the subsoil had a big effect on the Shannon, ACE, and Chao1 indices, while pH and total phosphorus in the topsoil had a big effect on the Shannon and ACE indices of soil fungi (Figure 5B, *p* < 0.05). Furthermore, subsoil available phosphorus significantly influenced the Shannon index of soil fungi (Figure 5B, *p* < 0.05). The plant Shannon index significantly influenced the ACE index of soil fungi, and BGB significantly affected both the ACE and Chao1 indices of soil fungi. However, neither the plant Shannon index nor aboveground biomass significantly affected soil fungi diversity (Figure 5B, *p* < 0.05).

### 3.3. Co-Occurrence Networks

**Figure 6:**
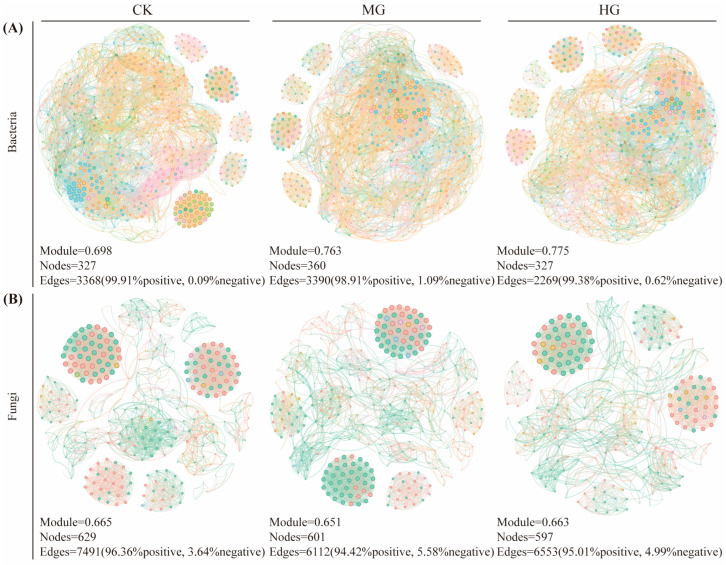
Co-occurrence network analysis of soil bacteria (**A**) and soil fungi (**B**) with relative abundance of OTUs greater than or equal to 0.1% under the different grazing intensities. The size of each node is proportional to the relative abundance of OTUs, and the colors of the nodes indicate different modularity classes. The edges indicate the significant Spearman correlations (|*r*| > 0.8 and *p* < 0.05). Null grazing, CK; moderate grazing, MG; and heavy grazing, HG.

Compared to the CK (327 nodes and 3368 edges), MG resulted in a more complex co-occurrence network in soil bacteria, featuring 360 nodes and 3390 edges. In contrast, there is a simpler co-occurrence network in soil bacteria under the HG condition with 327 nodes and 2269 edges (Figure 6A). MG and HG enhanced the network modularity of the soil bacterial co-occurrence network with modularity values of 0.698 for CK, 0.763 for MG, and 0.775 for HG. MG and HG also led to an increased number of negative connections within the networks, with 1.09% for MG and 0.62% for HG, relative to 0.09% for CK.

Compared to the soil bacterial co-occurrence network, the soil fungal co-occurrence network contained more nodes and edges, yet it formed a simpler structure (Figure 6B). The soil fungal co-occurrence networks under MG (601 nodes, 6112 edges, and 0.651 modularity) and under HG (597 nodes, 6553 edges, and 0.663 modularity) were simpler than those under CK (629 nodes, 7491 edges, and 0.665 modularity). Moreover, grazing led to a higher percentage of negative connections in the network, which was 5.58% for MG and 4.99% for HG, compared to 3.64% for CK.

**Figure 7:**
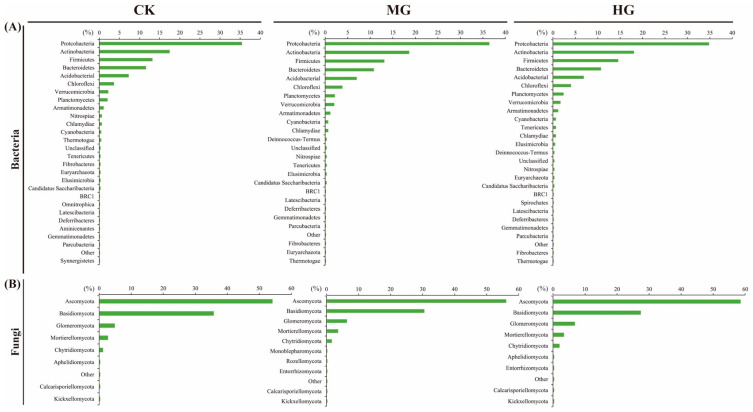
The proportion of phylum level of soil bacteria (**A**) and fungi (**B**) in the co-occurrence network under the different grazing intensities. Null grazing, CK; moderate grazing, MG; and heavy grazing, HG.

*Bacteroidetes*, *Acidobacteria*, and *Verrucomicrobia* made up less and less of the soil bacteria community as grazing got worse (Figure 7A). In contrast, the proportions of *Planctomycetes*, *Chloroflexi*, and *Armatimonadetes* among the soil bacterial community increased gradually with grazing intensity (Figure 7A). The proportions of *Proteobacteria* and *Actinobacteria* were highest under MG and lowest under HG (Figure 7A). Conversely, the proportion of *Firmicutes* was highest under HG and lowest under MG (Figure 7A). The proportions of *Ascomycota*, *Glomeromycota*, and *Chytridiomycota* among the soil fungal community increased with grazing intensity, whereas the proportion of *Basidiomycota* decreased (Figure 7B). The proportion of *Mortierellomycota* was also the highest under MG (Figure 7B).

## 4. Discussion

### 4.1. The Effect of Grazing Intensity on Microbial Diversity

Grazing intensity had an insignificant effect on the relative abundance of dominant bacterial and fungal phyla (Figure 1A,B). The dominant bacterial phyla—*Acidobacteria*, *Actinobacteria*, and *Proteobacteria*—remained stable across grazing intensities, consistent with findings from Rampelotto, et al. [33]. These phyla are widely distributed across various environments [34] and are highly prevalent in grasslands with or without grazing [11]. For instance, *Proteobacteria* are globally recognized as the most abundant microorganisms [34], while *Acidobacteria* and *Actinobacteria* are commonly found in arid and cold grasslands [35] and mountain meadows [36]. The ecological implications of these findings are significant. *Proteobacteria*, being the most dominant, play a crucial role in nitrogen fixation, converting atmospheric dinitrogen to ammonia and providing it to the host plant. This process is particularly important in grasslands where nitrogen is a limiting nutrient for plant growth. *Actinobacteria* contribute to nutrient enhancement, plant growth improvement, and phytopathogen inhibition, which can influence the overall health and productivity of the grassland ecosystem. Moderate grazing may increase bacterial diversity by promoting plant productivity and enhancing nutrient cycling, which facilitates processes like nitrogen mineralization, nitrification, and ammonification. This can lead to an increase in the relative abundance of certain bacterial phyla that are beneficial for plant growth and soil fertility. On the other hand, heavy grazing can disrupt plant communities and reduce soil carbon and nitrogen reserves, diminishing bacterial diversity. This is because HG can lead to soil compaction and reduced root exudation, which in turn affects the microbial communities that rely on these resources. For soil fungi, the dominant phyla—*Ascomycota*, *Basidiomycota*, and *Mortierellomycota*—were unaffected by grazing intensity, aligning with studies by Ren*,* et al. [37]. These phyla consistently dominate fungal communities across diverse habitats [38,39], attributed to their ability to produce protective conidia and adapt to adverse conditions through asexual reproduction [40,41]. The stability of these fungal phyla across grazing intensities suggests their resilience and adaptability to different management practices. However, the decline in fungal richness under HG, as indicated by lower ACE and Chao1 indices compared to CK and MG, is linked to reduced decomposition of plant litter and root exudates, limiting energy sources for fungi. Fungi play a critical role in the decomposition of organic matter and nutrient cycling, particularly in the formation of soil structure and the release of nutrients for plant uptake. The decrease in fungal diversity under HG could indicate a reduction in these ecosystem services, potentially affecting soil fertility and plant productivity in the long term.

Despite the stability in relative abundance, grazing intensity significantly influenced soil microbial diversity. The bacterial Shannon index increased under MG compared to CK, while HG had minimal effects (Figure 2A). This aligns with Zhou, et al. [42], who reported higher bacterial diversity under light and moderate grazing compared to heavy grazing or no grazing. MG promotes plant productivity and enhances nutrient cycling, facilitating processes like nitrogen mineralization, nitrification, and ammonification [43,44]. These processes are crucial for bacterial growth, as they increase the availability of nutrients required for bacterial metabolism and reproduction, thereby enhancing bacterial diversity. Additionally, MG can lead to increased plant diversity, which in turn supports a more diverse array of bacterial symbionts and decomposers [45]. Furthermore, the input of nutrients via dung and urine from livestock can alter the soil bacterial communities by providing additional resources [46], and changes in root exudates due to grazing can also influence the composition of bacterial communities [47] and modifications to soil texture [48]. Conversely, HG disrupts plant communities and reduces soil carbon and nitrogen reserves, diminishing bacterial diversity [49]. This is because HG can lead to soil compaction and reduced root exudation, in turn affecting the microbial communities that rely on these resources. For soil fungi, the ACE and Chao1 indices were significantly lower under HG compared to CK and MG, while the Shannon index remained unchanged (Figure 2B). This decline in fungal richness under HG is linked to reduced decomposition of plant litter and root exudates, limiting energy sources for fungi. Fungi are particularly sensitive to changes in soil structure and organic matter content, as they play a key role in the decomposition of more complex organic compounds. HG can lead to a reduction in plant litter, which is a primary substrate for fungal decomposition, thus reducing fungal diversity and complexity. Although some studies, such as that by Yin, et al. [50], suggest that fungal diversity decreases with grazing intensity, this variability may stem from differences in soil texture and local conditions. It is also possible that the grazing effects on fungi are mediated through changes in plant community composition, as plants can influence fungal communities through root exudates and litter quality.

Grazing intensity also distinctly impacted microbial community structure. MG significantly altered the bacterial community structure, while HG had a pronounced effect on fungal communities (Figure 3), consistent with Olivera, et al. [51]. The differential sensitivity of bacteria and fungi to environmental changes, resource competition, and nutrient depletion may explain why HG affects fungi more than bacteria. Bacteria, particularly phyla such as *Proteobacteria*, *Acidobacteria*, and *Actinobacteria*, are known for their adaptability and metabolic diversity. These groups are adept at rapidly colonizing disturbed habitats and utilizing newly available resources, such as those from nutrient inputs via dung and urine. The increased plant productivity under MG can enhance nutrient cycling, benefiting bacteria involved in processes like nitrogen mineralization, nitrification, and ammonification. This may explain why bacterial diversity can increase under moderate grazing conditions. In contrast, fungi, including *Ascomycota*, *Basidiomycota*, and *Mortierellomycota*, are often more sensitive to environmental changes due to their role in decomposing complex organic matter and their reliance on stable environments for spore germination and growth. Fungi have a greater ability to dissolve and utilize recalcitrant phosphorus than bacteria, and their utilization of phosphorus varies widely among different taxa. Under HG, the reduction in plant litter and root exudates can limit the energy sources available for fungi, leading to a decline in fungal richness. Additionally, fungi exhibit greater network modularity and more positive interactions than bacteria, implying that they have developed a stronger adaptive capability to cope with harsh environments [52,53,54]. However, when these environments are severely altered by HG, the fungal community structure is more drastically affected due to its reliance on specific conditions for its metabolic processes. The sensitivity of fungi to changes in soil nutrients and their role in nutrient cycling make them more vulnerable to the disruptions caused by HG. The decline in fungal diversity under HG could indicate a reduction in ecosystem services such as decomposition and nutrient cycling, potentially affecting soil fertility and plant productivity in the long term.

### 4.2. Influencing Factors of Microbial Diversity Under Different Grazing Intensities

Soil physicochemical factors regulate the alpha and beta diversity of soil microorganisms among grazing intensities. A correlation analysis showed a strong link between pH and fungal alpha diversity (Figure 4B), and a linear mixing effects model showed that soil pH significantly affects the Shannon and ACE indices of soil fungi (Figure 5B). Soil pH plays a significant role in regulating soil microbial diversity and community composition [26]. Griffiths, et al. [55] also found soil pH to be a key driver among the various factors influencing soil microbial communities. Soil pH directly impacts microbial growth and also indirectly modulates other co-varying factors that influence microbial diversity and community composition, including nutrient availability [56]. Additionally, soil total carbon significantly influences the ACE index of soil fungi (Figure 5B). This aligns with Fierer [57] and Delgado-Baquerizo, et al. [58], who emphasized the dual role of soil carbon as a nutrient source and a determinant of microbial diversity and community composition. Soil microorganisms reciprocally influence soil carbon cycling by facilitating organic carbon decomposition [56].

Plant diversity did not influence soil bacterial diversity, whereas belowground biomass had a pronounced effect on soil fungal diversity (Figure 4B). This is consistent with studies by Millard and Singh [59]; Wang, et al. [60]; and Pan, Peng, Wang, Tian, Zhang, Li, Yang, Wang, Chen and Niu [26], as they reported that plant diversity is not directly correlated with bacterial diversity but is significantly associated with fungal diversity. Alterations in plant community beta diversity can affect fungal community size and composition [61,62], likely due to fungi’s role as primary decomposers of lignocellulose, a vital carbon source for microorganisms [63]. Saprophytic fungi, for example, contribute significantly to carbon metabolism through enzymes involved in polymeric sugar and polysaccharide breakdown. Additionally, the unique mycelial structures and spores of fungi enhance their dependency on plants and their resilience to soil environment fluctuations [64]. Despite these findings, plant diversity and soil microorganisms maintain a complex relationship. Wang, Jiang, Struik, Wang, Jin, Wu, Na, Mu and Ta [10] demonstrated that bacterial and fungal alpha and beta diversities positively correlate with plant community diversity. However, bacterial community composition is more influenced by soil nutrients, while fungal composition is primarily driven by plant community composition [65]. Variations in plant community composition can selectively affect soil microorganisms through mechanisms like rhizodepositions, including root exudates, litter, and decaying roots [66]. The complex interplay between vegetation and environmental factors makes it challenging to isolate the effect of plant diversity on microbial communities in situ. In such ecosystems, both individual and combined factors significantly influence microbial systems [59]. Nevertheless, soil nutrients exert a stronger influence on microbial communities than plant composition [46]. Grassland management practices, such as grazing and fertilization, further modify microbial communities directly [67,68].

### 4.3. Effects of Different Grazing Intensities on Microbial Co-Occurrence Network

Microbial co-occurrence networks are non-random, covariant models that demonstrate how microbial communities respond to environmental factors and interact with living organisms [21]. Grazing intensity exerted distinct effects on microbial interactions (Figure 6A). The bacterial co-occurrence network was more intricate under MG and became simpler under HG compared to CK. This trend is attributed to two main factors. (1) Network complexity: MG increased the number of nodes and edges in the network, enhancing its modularization and connectivity. In contrast, HG simplified the network with fewer modules and weaker connections. (2) Dominant species dynamics: Grazing influenced the relative abundance of dominant bacterial taxa. Under MG, the proportions of *Proteobacteria* and *Actinobacteria* increased, while *Firmicutes* decreased. However, HG reversed this pattern, reducing *Proteobacteria* and *Actinobacteria* while increasing *Firmicutes* (Figure 7A). *Actinobacteria* thrive in nutrient-rich environments and play a crucial role in decomposing organic material [11,69]. MG likely benefited *Actinobacteria* by enhancing soil biomass and nutrients. Conversely, HG’s stress-induced conditions favored *Firmicutes*, particularly thick-walled bacteria known for their drought tolerance [70,71].

In contrast, fungal co-occurrence networks exhibited a different response. The fungal networks under MG and HG were simpler, with fewer modules than CK (Figure 6B). The modularity of fungal networks ranged from 0.651 to 0.665, lower than that of bacterial networks (0.698 to 0.775). Fungi’s unique biological characteristics, including slow growth rates, dormancy capabilities, and specialized cell membranes, enable them to withstand environmental fluctuations [72]. Grazing generally reduced fungal network complexity, but the functional implications remain unclear. Changes in fungal abundance at the phylum level provided further insights. As grazing intensity increased, the proportions of *Ascomycota*, *Glomeromycota*, and *Chytridiomycota* rose, while the proportion of *Basidiomycota* declined. This aligns with findings from Yang, Niu, Collins, Yan, Ji, Ling, Zhou, Du, Guo and Hu [11]; and Wang, Jiang, Struik, Wang, Jin, Wu, Na, Mu and Ta [10]. *Ascomycota*, particularly saprophytic fungi such as *Pezizaceae* and *Boudiera*, thrive in conditions with high levels of plant residues and animal feces [73]. Similarly, *Glomeromycota*, as arbuscular mycorrhizal fungi, form symbiotic relationships with plants, aiding in water and nutrient uptake under stress [10].

Grazing also increased negative interactions within microbial networks. For bacterial networks, the percentage of negative associations rose from 0.09% under CK to 1.09% under MG and 0.62% under HG. For fungal networks, negative associations increased from 3.64% (CK) to 5.58% (MG) and 4.99% (HG). These increases indicate intensified microbial competition under grazing conditions. Resource limitations in grasslands drive microbial species to compete for ecological niches, with more abundant communities engaging in greater competition [74]. However, variations in soil and plant types may moderate these interactions, as different microorganisms have varying adaptive capacities to specific environments [26,27,75].

## 5. Conclusions

This study has demonstrated that grazing intensity significantly influences soil microorganism alpha- and beta-diversities and their co-occurrence networks. Specifically, moderate grazing has been shown to increase bacterial diversity, alter community structure, and thereby enhance the complexity of co-occurrence networks, while heavy grazing decreases fungal diversity and alters community structure, leading to reduced network complexity. Plant diversity and belowground biomass emerged as primary factors influencing fungal diversity rather than bacterial diversity across different grazing intensities. In conclusion, moderate grazing appears to benefit alpine grassland biodiversity conservation, whereas heavy grazing is detrimental. Given the focus on Central Tibetan alpine grasslands, it is crucial to establish sustainable grazing intensities that consider the carrying capacity of alpine grasslands across various regions of Tibet. Our findings suggest that implementing grazing management practices that avoid overgrazing could play a significant role in preserving the biodiversity and ecological functions of alpine grasslands. Policymakers and land managers can use these insights to develop region-specific strategies that balance the economic benefits of livestock grazing with the need to maintain ecosystem health and resilience. Furthermore, future research should explore the development of advanced bioinformatics tools for the efficient filtering and merging of paired-end reads, which will enhance the accuracy and resolution of soil microbial community analyses. This will not only improve our understanding of the impacts of grazing practices but also contribute to the development of region-specific strategies for sustainable grazing management. By integrating our results with ongoing ecological and agricultural initiatives, we can work toward a more sustainable and ecologically responsible approach to grazing land management.

## Data Availability

The data presented in this study are available upon request from the corresponding author due to the fact that they are still being used for ongoing research.

## References

[1] Zhang Y., Ganjurjav H., Dong S.K., Gao Q.Z. (2020). Excessive plant compensatory growth: A potential endogenous driver of meadow degradation on the Qinghai-Tibetan Plateau. Ecosyst. Health Sustain..

[2] Qiu J. (2016). Trouble in Tibet. Nature.

[3] Yao T.D., Thompson L.G., Mosbrugger V., Zhang F., Ma Y.M., Luo T.X., Xu B.Q., Yang X.X., Joswiak D.R., Wang W.C. (2012). Third pole environment (TPE). Environ. Dev..

[4] Harris R.B. (2010). Rangeland degradation on the Qinghai-Tibetan plateau: A review of the evidence of its magnitude and causes. J. Arid. Environ..

[5] Chen S., Sun Y., Wang Y., Luo G., Ran J., Zeng T., Zhang P. (2024). Grazing weakens the linkages between plants and soil biotic communities in the alpine grassland. Sci. Total Environ..

[6] Xu X.F., Wang N.N., Lipson D., Sinsabaugh R., Schimel J., He L.Y., Soudzilovskaia N.A., Tedersoo L. (2020). Microbial macroecology: In search of mechanisms governing microbial biogeographic patterns. Glob. Ecol. Biogeogr..

[7] Liao H., Li C., Ai Y., Li X. (2023). Soil bacterial responses to disturbance are enlarged by altitude in a mountain ecosystem. J. Soils Sediments.

[8] Nottingham A.T., Fierer N., Turner B.L., Whitaker J., Ostle N.J., McNamara N.P., Bardgett R.D., Leff J.W., Salinas N., Silman M.R. (2018). Microbes follow Humboldt: Temperature drives plant and soil microbial diversity patterns from the Amazon to the Andes. Ecology.

[9] Delgado-Baquerizo M., Maestre F.T., Reich P.B., Trivedi P., Osanai Y., Liu Y.R., Hamonts K., Jeffries T.C., Singh B.K. (2016). Carbon content and climate variability drive global soil bacterial diversity patterns. Ecol. Monogr..

[10] Wang Z., Jiang S.Y., Struik P.C., Wang H., Jin K., Wu R.L.G., Na R.S., Mu H.B., Ta N. (2023). Plant and soil responses to grazing intensity drive changes in the soil microbiome in a desert steppe. Plant Soil.

[11] Yang F., Niu K.C., Collins C.G., Yan X.B., Ji Y.G., Ling N., Zhou X.H., Du G.Z., Guo H., Hu S.J. (2019). Grazing practices affect the soil microbial community composition in a Tibetan alpine meadow. Land Degrad. Dev..

[12] Luo S., Png G.K., Ostle N.J., Zhou H.K., Hou X.Y., Luo C.L., Quinton J.N., Schaffner U., Sweeney C., Wang D.J. (2023). Grassland degradation-induced declines in soil fungal complexity reduce fungal community stability and ecosystem multifunctionality. Soil. Biol. Biochem..

[13] Mueller P., Granse D., Nolte S., Do H.T., Weingartner M., Hoth S., Jensen K. (2017). Top-down control of carbon sequestration: Grazing affects microbial structure and function in salt marsh soils. Ecol. Appl..

[14] Zhang M., Delgado-Baquerizo M., Li G., Isbell F., Wang Y., Hautier Y., Wang Y., Xiao Y., Cai J., Pan X. (2023). Experimental impacts of grazing on grassland biodiversity and function are explained by aridity. Nat. Commun..

[15] Yang Y., Wu L., Lin Q., Yuan M., Xu D., Yu H., Hu Y., Duan J., Li X., He Z. (2013). Responses of the functional structure of soil microbial community to livestock grazing in the Tibetan alpine grassland. Glob. Chang. Biol..

[16] Treweek G., Di H.J., Cameron K.C., Podolyan A. (2016). Simulated animal trampling of a free-draining stony soil stimulated denitrifier growth and increased nitrous oxide emissions. Soil Use Manag..

[17] Kohler F., Hamelin J., Gillet F., Gobat J.M., Buttler A. (2005). Soil microbial community changes in wooded mountain pastures due to simulated effects of cattle grazing. Plant Soil.

[18] Xu H., You C., Tan B., Xu L., Liu Y., Wang M., Xu Z., Sardans J., Peñuelas J. (2023). Effects of livestock grazing on the relationships between soil microbial community and soil carbon in grassland ecosystems. Sci. Total Environ..

[19] Zhou J., Zhang M., Raza S.T., Yang S., Liu J., Cai M., Xue S., Wu J. (2023). Fungal but not bacterial β-diversity decreased after 38-year-long grazing in a southern grassland. Plant Soil.

[20] Xun W.B., Yan R.R., Ren Y., Jin D.Y., Xiong W., Zhang G.S., Cui Z.L., Xin X.P., Zhang R.F. (2018). Grazing-induced microbiome alterations drive soil organic carbon turnover and productivity in meadow steppe. Microbiome.

[21] Faust K., Raes J. (2012). Microbial interactions: From networks to models. Nat. Rev. Microbiol..

[22] Zhang B.G., Zhang J., Liu Y., Shi P., Wei G.H. (2018). Co-occurrence patterns of soybean rhizosphere microbiome at a continental scale. Soil Biol. Biochem..

[23] Nemergut D.R., Schmidt S.K., Fukami T., O’Neill S.P., Bilinski T.M., Stanish L.F., Knelman J.E., Darcy J.L., Lynch R.C., Wickey P. (2013). Patterns and processes of microbial community assembly. Microbiol. Mol. Biol. Rev..

[24] Jing L., Mipam T.D., Ai Y., Jiang A., Gan T., Zhang S., Liu J., Tian L. (2023). Grazing intensity alters soil microbial diversity and network complexity in alpine meadow on the Qinghai-Tibet Plateau. Agric. Ecosyst. Environ..

[25] Cornell C.R., Zhang Y., Ning D., Xiao N., Wagle P., Xiao X., Zhou J. (2023). Land use conversion increases network complexity and stability of soil microbial communities in a temperate grassland. ISME J..

[26] Pan J., Peng Y., Wang J., Tian D., Zhang R., Li Y., Yang L., Wang S., Chen C., Niu S. (2023). Controlling factors for soil bacterial and fungal diversity and composition vary with vegetation types in alpine grasslands. Appl. Soil Ecol..

[27] Gong J.R., Zhu C.C., Yang L.L., Yang B., Wang B., Baoyin T.T., Liu M., Zhang Z.H., Shi J.Y. (2020). Effects of nitrogen addition on above-and belowground litter decomposition and nutrient dynamics in the litter-soil continuum in the temperate steppe of Inner Mongolia, China. J. Arid. Environ..

[28] Ma C., Zhao T., Baoyin T., Han X., Frey B., Yang J., Dong S. (2024). Long-term grazing reduces soil fungal network complexity but enhances plant-soil microbe network connectivity in a semi-arid grassland. Sci. Total Environ..

[29] Guseva K., Darcy S., Simon E., Alteio L.V., Montesinos-Navarro A., Kaiser C. (2022). From diversity to complexity: Microbial networks in soils. Soil. Biol. Biochem..

[30] Xiang M.X., Wu J.X., Wu J.J., Guo Y.J., Lha D., Pan Y., Zhang X.Z. (2021). Heavy grazing altered the biodiversity–productivity relationship of alpine grasslands in Lhasa river valley, Tibet. Front. Ecol. Evol..

[31] Zhong Z., Fu G. (2022). Response of soil fungal species, phylogenetic and functional diversity to diurnal asymmetric warming in an alpine agricultural ecosystem. Agric. Ecosyst. Environ..

[32] Fu F., Li Y., Zhang B., Zhu S., Guo L., Li J., Zhang Y., Li J. (2024). Differences in soil microbial community structure and assembly processes under warming and cooling conditions in an alpine forest ecosystem. Sci. Total Environ..

[33] Rampelotto P.H., de Siqueira Ferreira A., Barboza A.D.M., Roesch L.F.W. (2013). Changes in Diversity, Abundance, and Structure of Soil Bacterial Communities in Brazilian Savanna Under Different Land Use Systems. Microb. Ecol..

[34] Ligi T., Oopkaup K., Truu M., Preem J.K., Nolvak H., Mitsch W.J., Mander U., Truu J. (2014). Characterization of bacterial communities in soil and sediment of a created riverine wetland complex using high-throughput 16S rRNA amplicon sequencing. Ecol. Eng..

[35] Maestre F.T., Delgado-Baquerizo M., Jeffries T.C., Eldridge D.J., Ochoa V., Gozalo B., Quero J.L., Garcia-Gomez M., Gallardo A., Ulrich W. (2015). Increasing aridity reduces soil microbial diversity and abundance in global drylands. Proc. Natl. Acad. Sci. USA.

[36] Gregus Z., Watkins J.B., Thompson T.N., Klaassen C.D. (1982). Resistance of some phase II biotransformation pathways to hepatotoxins. J. Pharmacol. Exp. Ther..

[37] Ren C., Zhang W., Zhong Z., Han X., Yang G., Feng Y., Ren G. (2018). Differential responses of soil microbial biomass, diversity, and compositions to altitudinal gradients depend on plant and soil characteristics. Sci. Total Environ..

[38] Beimforde C., Feldberg K., Nylinder S., Rikkinen J., Tuovila H., Dorfelt H., Gube M., Jackson D.J., Reitner J., Seyfullah L.J. (2014). Estimating the Phanerozoic history of the Ascomycota lineages: Combining fossil and molecular data. Mol. Phylogenet. Evol..

[39] Bai X., Zhang E., Wu J., Ma D., Zhang C., Zhang B., Liu Y., Zhang Z., Tian F., Zhao H. (2024). Soil fungal community is more sensitive than bacterial community to modified materials application in saline–alkali land of Hetao Plain. Front. Microbiol..

[40] Feofilova E.P., Ivashechkin A.A., Alekhin A.I., Sergeeva Y.E. (2012). Fungal spores: Dormancy, germination, chemical composition, and role in biotechnology (review). Appl. Biochem. Microbiol..

[41] Griffin D.H. (1996). Fungal Physiology.

[42] Zhou X.Q., Wang J.Z., Hao Y.B., Wang Y.F. (2010). Intermediate grazing intensities by sheep increase soil bacterial diversities in an Inner Mongolian steppe. Biol. Fert. Soils.

[43] Xu Y.Q., Li L.H., Wang Q.B., Chen Q.S., Cheng W.X. (2007). The pattern between nitrogen mineralization and grazing intensities in an Inner Mongolian typical steppe. Plant Soil.

[44] Brucek P., Simek M., Hynst J. (2009). Long-term animal impact modifies potential production of N_2_O from pasture soil. Biol. Fert. Soils.

[45] Dorrough J., Ash J., McIntyre S. (2004). Plant responses to livestock grazing frequency in an Australian temperate grassland. Ecography.

[46] Ritz K., McNicol J.W., Nunan N., Grayston S., Millard P., Atkinson D., Gollotte A., Habeshaw D., Boag B., Clegg C.D. (2004). Spatial structure in soil chemical and microbiological properties in an upland grassland. FEMS Microbiol. Ecol..

[47] Guitian R., Bardgett R.D. (2000). Plant and soil microbial responses to defoliation in temperate semi-natural grassland. Plant Soil.

[48] Chau J.F., Bagtzoglou A.C., Willig M.R. (2011). The Effect of Soil Texture on Richness and Diversity of Bacterial Communities. Environ. Forensics.

[49] Steffens M., Kolbl A., Totsche K.U., Kogel-Knabner I. (2008). Grazing effects on soil chemical and physical properties in a semiarid steppe of Inner Mongolia (PR China). Geoderma.

[50] Yin Y.L., Wang Y.Q., Li S.X., Liu Y., Zhao W., Ma Y.S., Bao G.S. (2019). Effects of enclosing on soil microbial community diversity and soil stoichiometric characteristics in a degraded alpine meadow. Ying Yong Sheng Tai Xue Bao.

[51] Olivera N.L., Prieto L., Bertiller M.B., Ferrero M.A. (2016). Sheep grazing and soil bacterial diversity in shrublands of the Patagonian Monte, Argentina. J. Arid. Environ..

[52] Li Y.M., Wang S.P., Jiang L.L., Zhang L.R., Cui S.J., Meng F.D., Wang Q., Li X.N., Zhou Y. (2016). Changes of soil microbial community under different degraded gradients of alpine meadow. Agric. Ecosyst. Environ..

[53] Zhang Y., Xin L., Zhang J., Yang D. (2010). Effects of grazing on the community structure of soil bacteria in Stipa baicalensis steppe. Chin. J. Ecol..

[54] Fan J., Zhang C., Jin H., Zhang J., Han G. (2021). Grazing accelerates labile and recalcitrant soil carbon loss driving by rare microbial taxa in a desert steppe. Land Degrad. Dev..

[55] Griffiths R.I., Thomson B.C., James P., Bell T., Bailey M., Whiteley A.S. (2011). The bacterial biogeography of British soils. Environ. Microbiol..

[56] Tveit A., Schwacke R., Svenning M.M., Urich T. (2013). Organic carbon transformations in high-Arctic peat soils: Key functions and microorganisms. ISME J..

[57] Fierer N. (2017). Embracing the unknown: Disentangling the complexities of the soil microbiome. Nat. Rev. Microbiol..

[58] Delgado-Baquerizo M., Oliverio A.M., Brewer T.E., Benavent-Gonzalez A., Eldridge D.J., Bardgett R.D., Maestre F.T., Singh B.K., Fierer N. (2018). A global atlas of the dominant bacteria found in soil. Science.

[59] Millard P., Singh B.K. (2010). Does grassland vegetation drive soil microbial diversity?. Nutr. Cycl. Agroecosyst..

[60] Wang C.W., Ma L.N., Zuo X., Ye X.H., Wang R.Z., Huang Z.Y., Liu G.F., Cornelissen J.H.C. (2022). Plant diversity has stronger linkage with soil fungal diversity than with bacterial diversity across grasslands of northern China. Glob. Ecol. Biogeogr..

[61] McCulley R.L., Burke I.C. (2004). Microbial community composition across the Great Plains: Landscape versus regional variability. Soil. Sci. Soc. Am. J..

[62] Grayston S.J., Campbell C.D., Bardgett R.D., Mawdsley J.L., Clegg C.D., Ritz K., Griffiths B.S., Rodwell J.S., Edwards S.J., Davies W.J. (2004). Assessing shifts in microbial community structure across a range of grasslands of differing management intensity using CLPP, PLFA and community DNA techniques. Appl. Soil Ecol..

[63] De Vries F.T., Bardgett R.D. (2012). Plant–microbial linkages and ecosystem nitrogen retention: Lessons for sustainable agriculture. Front. Ecol. Environ..

[64] Sun S., Li S., Avera B.N., Strahm B.D., Badgley B.D. (2017). Soil Bacterial and Fungal Communities Show Distinct Recovery Patterns during Forest Ecosystem Restoration. Appl. Environ. Microb..

[65] Guo Y., Hou L., Zhang Z., Zhang J., Cheng J., Wei G., Lin Y. (2019). Soil microbial diversity during 30 years of grassland restoration on the Loess Plateau, China: Tight linkages with plant diversity. Land. Degrad. Dev..

[66] Grayston S.J., Griffith G.S., Mawdsley J.L., Campbell C.D., Bardgett R.D. (2001). Accounting for variability in soil microbial communities of temperate upland grassland ecosystems. Soil. Biol. Biochem..

[67] Griffiths R.I., Bailey M.J., McNamara N.P., Whiteley A.S. (2006). The functions and components of the Sourhope soil microbiota. Appl. Soil Ecol..

[68] Marilley L., Vogt G., Blanc M., Aragno M. (1998). Bacterial diversity in the bulk soil and rhizosphere fractions of Lolium perenne and Trifolium repens as revealed by PCR restriction analysis of 16S rDNA. Plant Soil.

[69] Fierer N., Bradford M.A., Jackson R.B. (2007). Toward an ecological classification of soil bacteria. Ecology.

[70] Eldridge D.J., Delgado-Baquerizo M., Travers S.K., Val J., Oliver I., Kardol P. (2017). Do grazing intensity and herbivore type affect soil health? Insights from a semi-arid productivity gradient. J. Appl. Ecol..

[71] Zhou C., Ma Z., Zhu L., Xiao X., Xie Y., Zhu J., Wang J. (2016). Rhizobacterial Strain Bacillus megaterium BOFC15 Induces Cellular Polyamine Changes that Improve Plant Growth and Drought Resistance. Int. J. Mol. Sci..

[72] Shu W.S., Huang L.N. (2022). Microbial diversity in extreme environments. Nat. Rev. Microbiol..

[73] Hansen K., Lobuglio K.F., Pfister D.H. (2005). Evolutionary relationships of the cup-fungus genus Peziza and Pezizaceae inferred from multiple nuclear genes: RPB2, beta-tubulin, and LSU rDNA. Mol. Phylogenetics Evol..

[74] Lupatini M., Suleiman A.K.A., Jacques R.J.S., Antoniolli Z.I., de Siqueira Ferreira A.O., Kuramae E.E., Roesch L.F.W. (2014). Network topology reveals high connectance levels and few key microbial genera within soils. Front. Environ. Sci..

[75] Barcenas-Moreno G., Gomez-Brandon M., Rousk J., Baath E. (2009). Adaptation of soil microbial communities to temperature: Comparison of fungi and bacteria in a laboratory experiment. Glob. Chang. Biol..

